# An Economic Gap Between the Recommended Healthy Food Patterns and Existing Diets of Minority Groups in the US National Health and Nutrition Examination Survey 2013–14

**DOI:** 10.3389/fnut.2019.00037

**Published:** 2019-04-04

**Authors:** Victor Fulgoni, Adam Drewnowski

**Affiliations:** ^1^Nutrition Impact LLC, Battle Creek, MI, United States; ^2^Center for Public Health Nutrition, University of Washington, Seattle, WA, United States

**Keywords:** USDA Food Patterns, Vegetarian, US-style, Mediterranean, nutrient density, food prices, diet cost, minorities

## Abstract

The US Department of Agriculture (USDA) has identified three Healthy Food Patterns as ways to implement the 2015–2020 Dietary Guidelines for Americans. We estimated the daily cost of the Healthy Vegetarian, US-Style, and Mediterranean Food Patterns (at 2,000 kcal/d) using national food prices adjusted for inflation. We also estimated the cost of existing dietary intakes in $/2,000 kcal/d for persons ≥2 years in the National Health and Nutrition Examination Survey (NHANES 2013–2014) using the same national food prices. The Nutrient Rich Food index (NRF9.3) was used as a measure of diet quality. Compared to existing diets, the USDA Healthy Food Patterns were higher in protein, fiber, vitamins, and minerals; were lower in solid fats, sugars, and sodium, and had higher diet quality scores. However, they also cost more. The cost of existing diets in NHANES was $5.47/d for Hispanics, $5.48/d for African-Americans, $5.94/d for Whites and $6.57/d for Asians. By contrast, the recommended US-Style Pattern cost $8.27/d, the Vegetarian Pattern cost $5.90/d, and the Mediterranean Pattern cost $8.73/d. Further, the Healthy Food Patterns featured some of the recommended food groups in unrealistic amounts. To ensure that the US Dietary Guidelines are both feasible and relevant to minority health, economic modeling studies should accompany government-issued dietary advice.

## Introduction

The 2015–2020 edition of the Dietary Guidelines for Americans (1) prominently featured three Healthy Food Patterns as prime examples of eating healthy (1). The Healthy Vegetarian Pattern, the Healthy US-Style Pattern, and the Healthy Mediterranean-Style Pattern were designed to show that the Dietary Guidelines could be met in a variety of ways (1). The amounts of different food groups to be included in the USDA Healthy Food Patterns were specified and limits were placed on the amounts of solid fats, added sugars, and alcohol (1). The recommended foods were to be in their nutrient-rich forms, low in sodium and with no added sugar (1).

The recommended USDA Healthy Food Patterns are a prominent tool of federal food and nutrition policy. Their estimated monetary cost in relation to the existing US diets has yet to be addressed. Yet there are well-documented inequities in access to affordable healthy foods across population subgroups in the US (2–4). Despite decades of dietary advice, the US diet continues to be high in calories, refined grains, added sugars, sodium, and saturated fat and remains low in whole grains, vegetables, and fruit (5, 6). Based on US Government reports, few, if any, improvements in diet quality have been observed in recent years (2). In general, African Americans and Hispanics consumed lower quality diets as compared to non-Hispanic Whites (5, 6).

For many low-income families, the additional cost of eating healthy may represent a genuine barrier to the adoption of government issued dietary guidance (3, 4). Ideally, Healthy Food Patterns ought to be affordable as well as nutrient-rich (7, 8). Early studies on the relation between US diet quality and its cost merged dietary recall data from the nationally representative National Health and Nutrition Examination Survey (NHANES) with mean national food prices issued by the USDA Center for Nutrition Policy and Promotion (9). Attaching national food prices to dietary recall data has become a standard practice for estimating daily diet costs at the individual level (9, 10).

In this study, existing diets from the 2013–14 National Health and Nutrition Examination Survey (2013–14 NHANES) (11) by race/ethnicity were compared to the recommended USDA Healthy Food Patterns (12). The comparisons were made in terms of nutrient density, calories from different food groups, and monetary cost per 2,000 kcal. The present hypothesis was that the USDA Healthy Food Patterns would be more nutrient-rich but also more costly as compared to the existing diets in the NHANES database. A secondary hypothesis was that the gaps in diet quality and cost between recommendations and reality would be highest for African-American and Hispanic minority groups (3–5).

## Materials and Methods

### The National Health and Nutrition Examination Survey (2013–2014 NHANES)

What We Eat in America is the dietary intake interview component of the National Health and Nutrition Examination Survey (NHANES). Dietary intake data for the present analyses came from the first day 24-h recall in 2013–2014 NHANES for all participants aged >2 years (*N* = 8.062), and excluding pregnant or lactating females. The 24-h recall data includes the amount in grams and a description of each individual food and beverage consumed from midnight to midnight on the previous day. The examination protocol, data collection methods, and quality controls for each 24-h recall have been documented elsewhere (11). The present analyses were based on 7,967 persons aged >2 years.

Information from the demographic NHANES questionnaires was used to stratify the sample by race/ethnicity, defined as Hispanic, African American (Black), White, Asian, and Other (11). Analyses of publicly available federal NHANES database are exempt from approvals by Institutional Review Boards.

### The USDA Healthy Food Patterns

The USDA constructed Healthy Food Patterns at 12 energy levels, ranging from 1,000 to 3,200 kcal (1). Table 1 shows the recommended amounts of food from each food group for Healthy Food Patterns at the 2,000 kcal/d energy level (1, 12). Nutrient and energy contributions from each group were calculated based on the nutrient-dense foods in each group (e.g., lean meats and fat-free milk). Nutrient-dense forms of food were defined as being lean or low-fat and prepared without added fats, sugars, refined starches, or salt. The number of calories from added sugars and solid fats that could be accommodated within each energy level was specified as well. Those calories, mostly from added sugars and fat are indicated in Table 1 as “other” calories (1). Additional documentation for the USDA Healthy Food Patterns and the Recommended Intake Amounts is provided online (1).

**Table 1:** Recommended amounts of food from each food group at 2,000 kcal/d energy level by USDA Healthy Food Pattern.

**Food Group**	**Vegetarian**	**Healthy US-Style**	**Mediterranean**
Vegetables (c-eq/day)	2^1/2^	2^1/2^	2^1/2^
Dark green vegetables (c-eq/wk)	1^1/2^	1^1/2^	1^1/2^
Red and orange vegetables (c-eq/wk)	5^1/2^	5^1/2^	5^1/2^
Legumes (beans and peas) (c-eq/wk)	1^1/2^	1^1/2^	1^1/2^
Starchy vegetables (c-eq/wk)	5	5	5
Other vegetables (c-eq/wk)	4	4	4
Fruits (c-eq/day)	2	2	2^1/2^
Grains (oz-eq/day)	6^1/2^	6	6
Whole grains (oz-eq/day)	3^1/2^	3	3
Refined grains (oz-eq/day)	3	3	3
Dairy (c-eq/day)	3	3	2
Protein foods (oz-eq/day)	3^1/2^	5^1/2^	6^1/2^
Seafood (oz-eq/wk)	0	8	15
Meats, poultry, eggs (oz-eq/wk)	3 (eggs)	26	26
Nuts, seeds, soy (oz-eq/wk)	7 (nuts, seeds) + 8 (soy)	5	5
Oils (g/day)	27	27	27
Limits on other kcal (%)	290 (15%)	270 (14%)	260 (13%)

*Adapted from 2015 to 2020 Dietary Guidelines for Americans (1)*.

The pricing strategy was based on the published USDA directives for constructing the Healthy Food Patterns. Based on USDA guidelines, the foods selected for pricing the Healthy Food Patterns were therefore (a) nutrient-rich versions, within each food group (b) low in sodium, based on the sodium content per 100 g, and (c) containing no added sugar. The USDA guidelines specifically stated that only the lower sodium foods should be chosen. The cutoff points for the selective inclusion of lower sodium foods removed foods with the highest sodium content, while allowing the widest range of qualifying food choices. Additional criteria included placing limits on saturated fat and cholesterol (for meat, poultry and eggs). Analyses used the 2013–2104 Food and Nutrient Database for Dietary Studies (2013–14 FNDDS) and the Food Patterns Equivalents Database (FPED) (12). The aggregation and exclusion codes used for constructing the nutrient rich food groups database are provided in the Appendix.

The energy and nutrient contribution of individual foods to each food group was based on the amounts consumed by all participants aged >2 years in the 2013–2014 NHANES. For example, food items within the dark green vegetable category were weighted by amounts consumed (in g) to arrive at a composite nutrient profile. At this time, the weighting was not stratified by age group, gender, or race/ethnicity.

### The USDA National Food Prices Database

The USDA national food prices database, derived from the Nielsen Homescan Consumer Panel price data and released in May 2008, provided national food prices for all foods in 2001–2004 NHANES (9). The food and beverage prices were computed by the USDA per gram edible portion ($/g, edible portion), adjusting for preparation losses and gains (9).

The present adjustment of 2001–04 prices for inflation was based on the Consumer Price Index data from the Bureau of Labor Statistics (BLS) (13) and was guided by previously published work (5). The BLS publishes the Consumer Price Index (CPI) month-by-month for multiple food categories. The 2001–2004 FNDDS food codes were mapped to the BLS series in order to adjust the 2001–2004 food prices to the period 2013–2014. This required matching of any new food codes for 2013–14 with the most closely matching food codes in the 2001–2004 USDA Food and Nutrient Database for Dietary Studies. Mixed dishes, composed of multiple ingredients, that did not map to a single BLS series were regressed on the Food Patterns Equivalents Database (FPED) food components (12). The regression coefficients were then applied to the food components of mixed dish food codes to obtain CPI values. Consumer price indexes for a 2-years NHANES cycle were obtained by averaging the BLS monthly values over the 2 years cycle.

In analyses of 2013–14 NHANES data, the cost vector was essentially treated as another nutrient. The cost per 100 g edible portion of each food or beverage was summed across all foods consumed by that individual. The estimated monetary costs of existing diets were calculated for each racial/ethnic group and standardized to 2,000 kcal. Those data were expressed as means and standard errors (SEMs).

### The Calculated Monetary Cost of USDA Healthy Food Patterns

The fixed cost of the USDA Healthy US-Style, Healthy Vegetarian and Healthy Mediterranean Food Patterns was estimated based on the amounts listed for 2,000 kcal/d. The recommended amounts were converted to grams and were multiplied by the mean price per gram for each qualifying food group. The costs were summed for each food group and for each Food Pattern.

For purposes of modeling average prices of each food group, qualifying foods within each food group/subgroup were assumed to be consumed in the same amounts as that listed in the individual foods file in the NHANES dietary intakes database.

**Figure 1:**
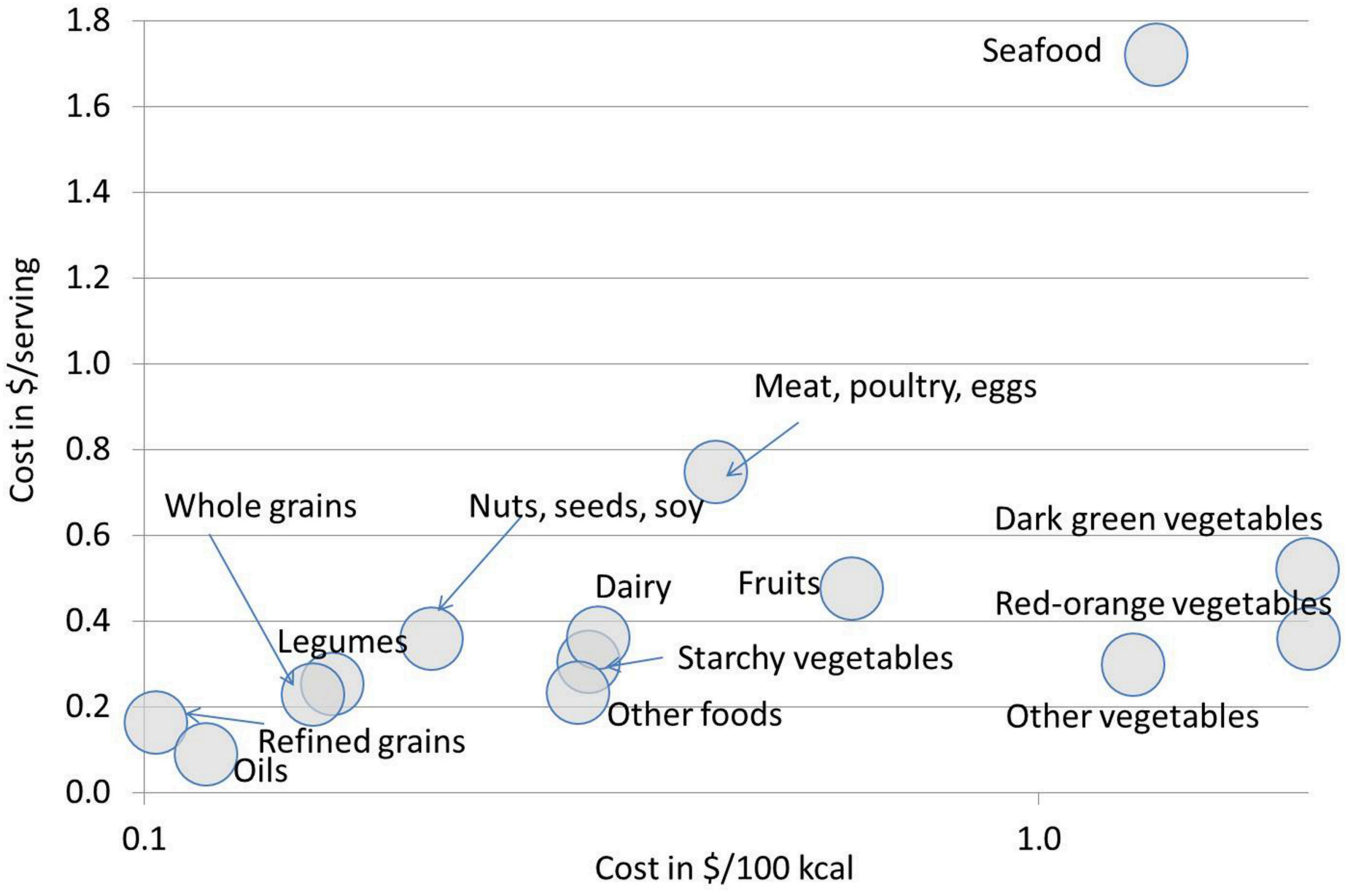
Mean costs, in 2013–14 US prices, for the USDA Healthy Pattern food categories. Shown are data in $/100 kcal (x-axis; logarithmic scale) and $/serving (y-axis).

The present cost analyses included fresh, frozen, and canned foods in proportion to their consumption patterns in the NHANES sample. Similar procedures were followed for the costing of the Mediterranean and Vegetarian Food Patterns, again following the USDA specifications and requirements. Figure 1, a semi-log scatterplot, shows the mean costs (per 100 kcal and per serving) for each food group. As expected, grains, oils and legumes were substantially cheaper per 100 kcal than meat, poultry and eggs, fruits, seafood, and vegetables. The goal was not to find the lowest cost healthy choices, but rather to estimate the likely cost of selecting one of the USDA Healthy Food Patterns, while staying within the eating habits of the US population.

### The Nutrient Rich Food Diet Quality Index

Calculations of the Nutrient Rich Food index, a measure of dietary nutrient density, were based on published work (14). The composite Nutrient Rich Food (NRF9.3) index was the sum of percent daily values (%DVs) for nine qualifying nutrients, minus the sum of %DVs for three disqualifying nutrients. The positive Nutrient Rich (NR) subscore was based on nine qualifying nutrients: protein, fiber, vitamins A, C, and D, calcium, iron, potassium, and magnesium. The negative Limiting Nutrients (LIM) subscore was based on three disqualifying nutrients: saturated fat, added sugar, and sodium (14). Nutrient standards, provided by the Food and Drug Administration and the National Academy of Sciences, were used to calculate the NR9 and the LIM subscores are shown in Table 2 (15, 16). The NRF9.3 algorithm was applied to the total population diet in the 2013–14 NHANES. For the present version of the NRF9.3, the Daily Values for nutrients were not capped at 100% DV.

**Table 2:** Nutrient standards for calculating the Nutrient Rich Foods index NRF9.3_ a measure of nutrient density based on 2,000 kcal/day diet.

**Positive NR9 subscore**		**Negative LIM subscore**	
Nutrient	Reference DV	Nutrient	MRA
Protein	50 g	Saturated fat	20 g
Fiber	25 g	Added sugars	50 g (12.5 tsp)
Vitamin A	1,515 mcg	Sodium	2,400 mg
Vitamin C	60 mg	–	–
Vitamin D	15 IU	–	–
Calcium	1,000 mg	–	–
Iron	18 mg	–	–
Potassium	3,500 mg	–	–
Magnesium	400 mg	–	–

### Statistical Analyses

Data analyzed were the first day of NHANES 2013–14 dietary intakes for persons >2 years, excluding pregnant or lactating women. Analyses used SAS 9.2 together with dietary survey weight. SAS codes used to define qualifying foods in their nutrient dense form are listed in Appendix Table A1. Comparisons of the total cost of existing diets by race/ethnicity were based on *t*-tests adjusted for multiple comparisons.

## Results

### The USDA Healthy Food Patterns Featured More Recommended Food Groups

**Figure 2:**
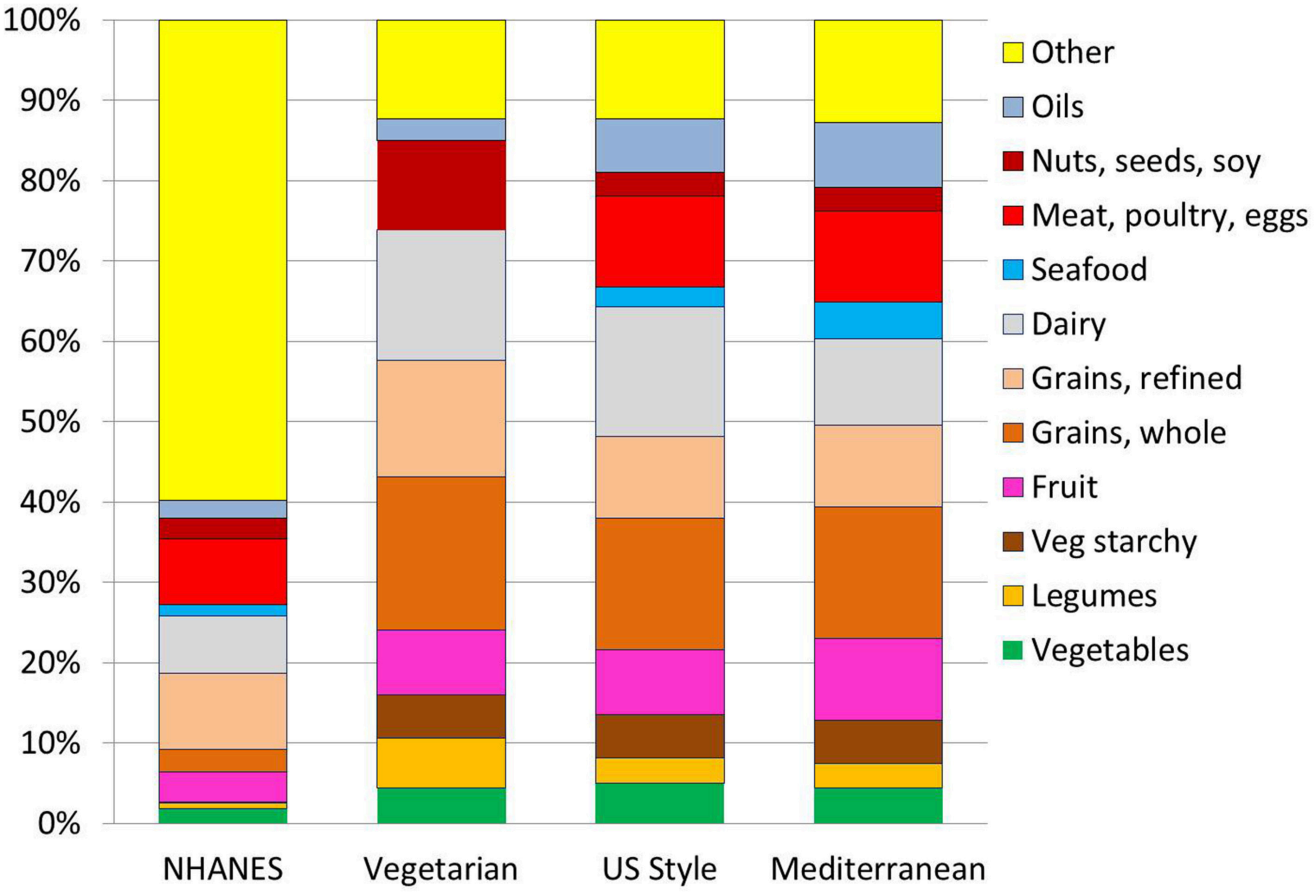
Diet composition in %kcal/d from each food groups for each of the USDA Healthy Food Patterns. The comparison is betwen an existing diet for persons >2 years in NHANES 2013–14 and the recommended USDA Healthy Vegetarian, Healthy US-Style and Healthy Mediterranan. For the Healthy Vegetarian Food Pattern, calories from eggs were included with nuts, seeds, and soy.

Figure 2 shows energy contribution (in percent kcal/d) of each food group to the existing NHANES 2013–14 diets and to the recommended USDA Healthy Food Patterns. While the existing NHANES diets derived the bulk of dietary energy from “other” foods, the USDA Healthy Food Patterns featured more vegetables (including starchy) and legumes; more fruit and whole grains; more low fat dairy, lean meats and seafood. Instead of meat and seafood, the Vegetarian Pattern featured sharply higher amounts of legumes and a sharp increase in soy. The data are expressed as percent kcal in 2,000 kcal/d diet.

Additional analyses by food weight (data in Appendix Table A2) showed that low-fat dairy products measured in cup-equivalents increased from 1.7 cup-eq. in existing diets to a recommended 3.1 (US-Healthy); 3.2 (Vegetarian), and 2.1 (Mediterranean). Amounts of yogurt increased 3-fold (0.06–0.2), but not for the Mediterranean Pattern (0.13). Seafood went from existing 0.43 to 1.29 oz-eq (Mediterranean). Amounts of lean meat went up for the US-Healthy and Mediterranean Patterns.

The prescribed amounts of vegetables (in cup-equivalents) more than doubled from the existing 1.3 to a recommended 2.91 (US-Healthy). Amounts of dark green vegetables tripled from the existing 0.13 to a recommended 0.37 (US-Healthy); red and orange vegetables doubled from the existing 0.27 to recommended 0.56 (US and Mediterranean); as did starchy vegetables (0.4–0.9).

The prescribed amounts of whole fruit (also in cup-equivalents) quadrupled from 0.63 in existing diets to a recommended 2.5 (Mediterranean). Fruit juices (100%) were increased by 25% for US-Healthy and Mediterranean Patterns (from 0.28 to 0.4) but showed a sharp drop for the Vegetarian Pattern as compared to existing NHANES diets.

Whole grains (in cup equivalents) increased more than 4-fold, from the existing 0.81 to recommended 3.71 (Vegetarian), whereas refined grains were cut by half, from the existing 5.5 cup-eq to recommended 2.5 (US-Healthy and Mediterranean). Nuts and seeds were increased for the Vegetarian Pattern (0.7–1.1) but reduced to 0.4 for both US-Healthy and Mediterranean Patterns, as compared to existing NHANES diets.

### The USDA Healthy Food Patterns Had Higher NRF9.3 Scores

**Figure 3:**
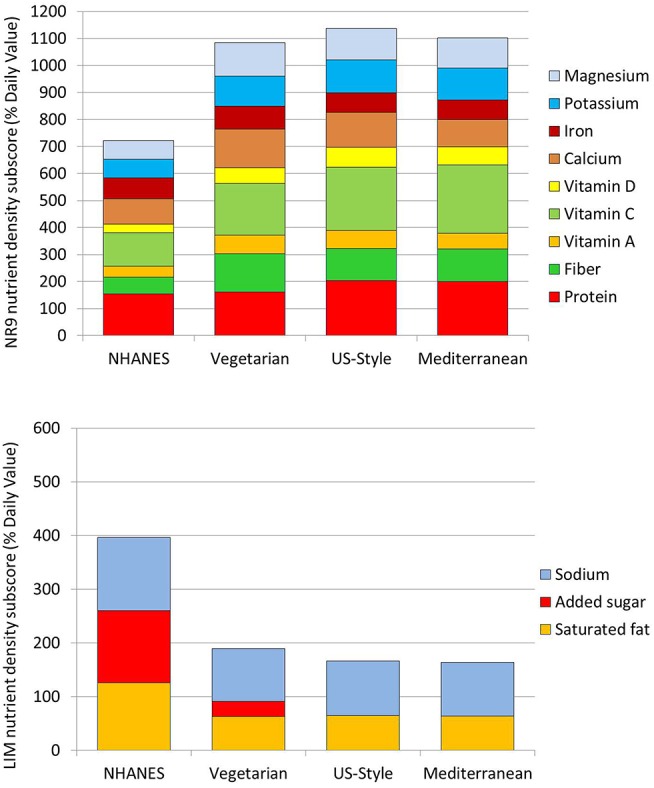
Nutrient density sub-scores for 9 qualifying nutrients and for 3 limiting nutrients. The Nutrient Rich subscore (NR) for qualifying nutrients is shown in the top panel; the Limiting subcore (LIM) is shown in the bottom panel. The comparison is between existing diets for persons >2 years in NHANES 2013–14 and the recommended USDA Healthy Food Patterns: Healthy Vegetarian, Healthy U.S.-Style, and the Healthy Mediterranean.

Figure 3 shows the Nutrient Rich (NR9) and the Limiting Nutrients (LIM) subscores for the NRF nutrient density model. The NR9 subscores for the recommended USDA Healthy Food Patterns were uniformly higher as compared to existing NHANES diets. As shown in Figure 3 (top), the recommended Healthy Food Patterns had more protein, fiber, vitamins, and minerals. Additional analyses (data not shown) indicated that protein increased from a mean of 77.2 g/d in existing NHANES diets to a recommended 102.2 g/d (US-Healthy), or about 200% DV. Fiber increased from 15.5 g/d in existing diets to a recommended 30.3 g/d (Mediterranean) and 35.5 g/d (Vegetarian). Vitamin C increased from 74 mg/d in existing diets to a recommended 151 mg/d (Mediterranean), or about 250% DV.

As shown in Figure 3 (bottom) the LIM subscores for the Healthy Food Patterns were sharply lower. In particular, added sugars were reduced to zero in the recommended US-Healthy and Mediterranean Patterns.

### The USDA Healthy Food Patterns Had Higher Monetary Costs

Table 3 shows estimated diet costs in $/2,000 kcal/d of existing diets and dietary components in the National Health and Nutrition Examination Survey (NHANES 2013–14) by race/ethnicity. Data are for persons >2 years with exclusions for pregnant or lactating females. Presented are means and standard errors (SEM). The average diet cost for persons >2 years was estimated at $5.82, adjusted to 2,000 kcal/d. The estimated daily diets costs by racial/ethnic groups were: $5.48/d (Blacks), $5.47/d (Hispanics), $5.94/d (Whites), $6.57/d (Asians), and $5.51 (Other). In univariate statistics, total diets costs for Black and Hispanic groups were not significantly different form each other but were significantly below those for Whites (*p* < 0.001). Asians' diet costs were significantly above the other four groups (*p* < 0.01).

**Table 3:** Estimated diet costs in $/2,000 kcal/d of existing diets and dietary components in the National Health and Nutrition Examination Survey (NHANES 2013–14) by race/ethnicity.

	**All (*****N*** **=** **7,967)**		**Hispanic (*****N*** **=** **2,106)**		**Black (*****N*** **=** **1,743)**		**White (*****N*** **=** **3,002)**		**Asian (*****N*** **=** **773)**		**Other (*****N*** **=** **343)**	
***N* =**	**Mean**	**SEM**	**Mean**	**SEM**	**Mean**	**SEM**	**Mean**	**SEM**	**Mean**	**SEM**	**Mean**	**SEM**
Vegetables, dark green	0.04	0.00	0.02	0.00	0.05	0.01	0.04	0.01	0.08	0.01	0.02	0.01
Vegetables, red, and orange	0.06	0.00	0.05	0.00	0.04	0.01	0.07	0.00	0.06	0.01	0.05	0.01
Legumes (beans and peas)	0.02	0.00	0.05	0.00	0.03	0.01	0.01	0.00	0.04	0.01	0.02	0.01
Starchy vegetables	0.01	0.00	0.00	0.00	0.01	0.00	0.00	0.00	0.00	0.00	0.00	0.00
Other vegetables	0.16	0.01	0.13	0.01	0.10	0.01	0.18	0.01	0.15	0.02	0.11	0.02
Fruits	0.38	0.01	0.42	0.03	0.31	0.02	0.36	0.01	0.60	0.03	0.35	0.05
Grains, whole	0.09	0.00	0.06	0.01	0.06	0.00	0.09	0.01	0.11	0.01	0.10	0.01
Grains, refined	0.20	0.00	0.18	0.01	0.19	0.01	0.20	0.01	0.29	0.03	0.17	0.02
Dairy	0.41	0.01	0.36	0.02	0.25	0.01	0.47	0.01	0.39	0.03	0.32	0.04
Seafood	0.30	0.04	0.22	0.04	0.44	0.08	0.27	0.04	0.55	0.08	0.46	0.22
Meat, poultry, eggs	0.70	0.03	0.79	0.04	0.81	0.06	0.66	0.03	0.78	0.07	0.57	0.07
Nuts, seeds, soy	0.10	0.01	0.07	0.01	0.06	0.01	0.11	0.01	0.14	0.02	0.12	0.04
Oils	0.04	0.00	0.03	0.00	0.04	0.00	0.05	0.00	0.03	0.00	0.03	0.00
Other	3.30	0.05	3.07	0.07	3.09	0.09	3.41	0.07	3.33	0.13	3.20	0.23
Total cost in $/d	5.82	0.07	5.47	0.11	5.48	0.16	5.94	0.09	6.57	0.20	5.51	0.37

*Data are for persons >2 years with exclusions for pregnant or lactating females*.

*Presented are means and standard errors (SEM)*.

**Figure 4:**
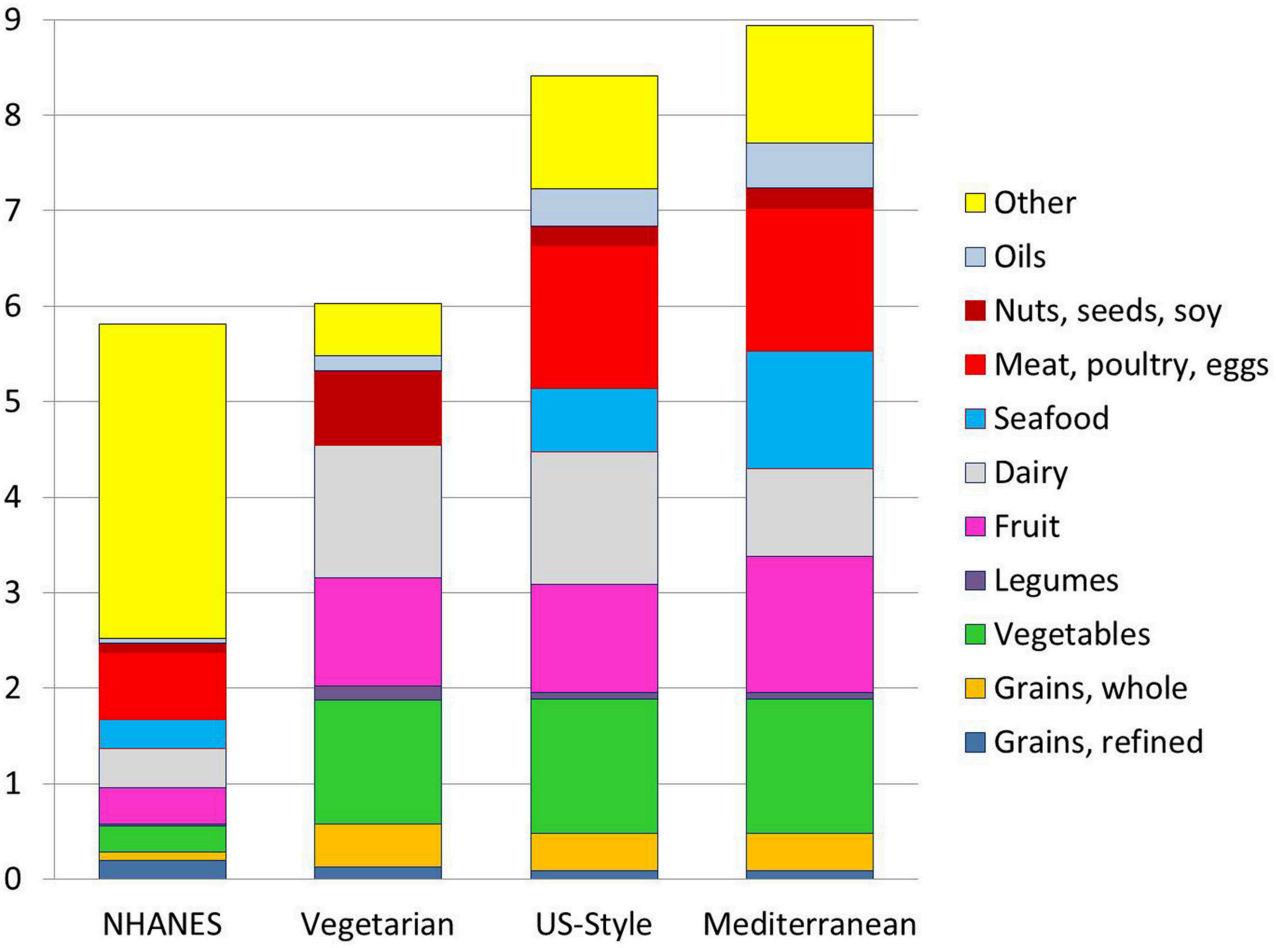
Estimated monetary costs in $/day by food group for a 2,000 kcal/d diet from NHANES 2013–14 as compared to the USDA Healthy Food Patterns. For the Healthy Vegetarian Food Pattern, calories from eggs were included with nuts, seeds, and soy.

Figure 4 shows the monetary costs of the recommended USDA Healthy Food Patterns. The calculated costs of Healthy Food Patterns were $5.90/d (Vegetarian); $8.27/d (US-Healthy), and $8.73/d (Mediterranean), all at 2,000 kcal/d.

## Discussion

Compared to existing NHANES diets, the recommended USDA Healthy Food Patterns were more nutrient-rich. All three recommended USDA Healthy Food Patterns were higher in protein, fiber, vitamins, and minerals and lower in saturated fat, added sugar, and sodium as compared to existing NHANES diets. Fully consistent with the 2015 Dietary Guidelines for Americans, the USDA Healthy Food Patterns also featured sharply higher amounts of the recommended food groups, while minimizing fat, sugar, and salt. As a result, the three USDA Healthy Food Patterns had higher NRF9.3 nutrient density scores than did the existing diets.

Not surprisingly, the USDA Healthy Food Patterns were also more costly per 2,000 kcal. That the cost of the recommended USDA Healthy Food Patterns was above that of existing NHANES diets was not a surprise. Consistent with multiple past studies, diets built around seafood, lean meats and poultry, dark green and orange-red vegetables, and whole fruit, were associated with higher per calorie diet costs (4).

The USDA Healthy Food Patterns were very high in some nutrients. Protein intakes were in excess of 200% DV (lower for Vegetarian). Vitamin C intakes were around 250% DV, far more than required. Based on statements from the National Academies (16), there are no recognized health benefits of usual protein, vitamin, or mineral intakes far in excess of the DRIs. While excess nutrients provide little or no health benefit, they can have major implications for raising diet cost.

The fact that all three Healthy Food Patterns were built around nutrient-rich foods most likely increased the prices even more. Selecting foods that have no added sugar and are low in saturated fat and salt is likely to add to food pattern costs. The Vegetarian Pattern benefitted from very large amounts of potatoes, legumes and soy and no seafood or meat. Fruit and dairy accounted for most of the cost. By contrast, the much higher costs of the US-Healthy and Mediterranean Patterns appeared to be driven by seafood, meat and poultry, fruit, and low-fat dairy. The high cost of the Mediterranean-Style Pattern was partly due to the high cost of seafood.

Some recommended food groups were provided in seemingly unrealistic amounts. For example, the recommended amounts of soy were increased 16-fold from existing levels, a 1600% increase. Conversely, some commonly eaten foods or nutrients (refined grains, added sugar) were reduced by 50% or eliminated altogether. When it comes to implementing public policy, food patterns that deviate greatly from current population eating habits have little chance of success (3, 17, 18). Ideally, healthy food patterns should provide adequate nutrition at low cost, while respecting existing dietary habits (e.g., Thrifty Food Plan) (19, 20). Making healthier foods more affordable through a variety of economic interventions ought to be at the center of dietary guidance (21–23).

The gap between the cost of existing diets and the cost of USDA Healthy Food Patterns was highest for African-American and Hispanic population subgroups, followed by Whites and Asians. The present analyses pointed to sharp disparities in diet quality and diet cost by racial/ethnic minority group in the 2013–14 NHANES data. Whereas, the USDA Healthy Food Patterns represent a desirable ideal, they are not equally realistic or equally attainable by every population subgroup. For some vulnerable groups, the economic gap may be too great (24). Based on the present estimations, replacing existing eating habits with the Healthy Mediterranean Pattern would lead to an average increase in daily diet cost of $1,062 per person per year. Put in a different way, each additional dollar spent on food per day represents 3% of income for a person at federal poverty level in the US ($12,140 in 2018).

Each food group can have its low cost and higher cost options. Our use of mean prices by food group prices did not allow us to price the lowest-cost version of the Mediterranean Food Pattern that could potentially be available to the low-income consumer. However, diet optimization studies (25) have shown that improving diet quality without increasing diet cost may require tolerating considerable deviance from existing eating habits (26).

The study had several limitations. First, analyses of existing diets were based on the first 24 h recall in the NHANES dietary intakes database. One day intake may not give a true picture of usual eating habits. Second, attaching retail prices to dietary intakes provides estimates of the monetary cost of each diets and not true food expenditures. In creating the prices database, the CNPP had been forced to assume that all foods were purchased at retail and prepared at home (9). Restaurant prices were not included. Another limitation was that the USDA food price database was based on a national panel and did not reflect local or regional differences. Additionally, the adjustment for inflation, based on BLS food categories, would not have captured price differentials within a category. However, the USDA used very similar methods to show that inflation-adjusted prices for fresh fruits and vegetables, eggs, red meat and cereals have risen whereas prices for fats and oils and sugar and sweets have fallen (27).

Finally, the estimated costs were based on the USDA mean prices by food category. Within-category food prices can be more or less expensive than the category mean. Even so, the present approach can give us insights into the relative affordability of nutritious diets and aid in understanding potential economic barriers to healthy eating.

## Conclusions and Implications

Behavioral and economic feasibility analyses can help DGAs to identify food patterns that are nutrient-rich, affordable, and appealing. Based on present analyses, the cost of the USDA Healthy Food Patterns exceeded that of existing NHANES diets, particularly as consumed by minority groups. Furthermore, some foods were proposed in amounts vastly different from the existing eating habits. For maximum efficacy, the recommended Healthy Food Patterns should be cost neutral (28). Future editions of the DGAs should consider affordability as a key component of the recommended Healthy Food Patterns, especially in the context of minority health.

## Data Availability

The datasets for this study will not be made publicly available because the NHANES datasets are publicly available and can be downloaded.

## Author Contributions

VF and AD conceptualized the study. VF conducted data analyses. AD took the lead on manuscript preparation; both authors reviewed, edited, and finalized the manuscript.

### Conflict of Interest Statement

VF is employed by Nutrition Impact LLC. AD has received grants, honoraria, and consulting fees from numerous food, beverage, and ingredient companies and other commercial and non-profit entities with an interest in the nutrient density of foods. The University of Washington receives research funding from public and private sectors.
