# Supplementary material for: An Economic Gap Between the Recommended Healthy Food Patterns and Existing Diets of Minority Groups in the US National Health and Nutrition Examination Survey 2013–14
Source: Front Nutr. 2019 Apr 4;6:37. doi: 10.3389/fnut.2019.00037 (PMC6458255; doi:10.3389/fnut.2019.00037)
Supplement: Supplementary file 1 [file Data_Sheet_1.docx]

**APPENDIX**: Food groups, Recommended Intakes and What We Eat in America (WWEIA) aggregation codes for the pricing of “nutrient rich foods”, per USDA requirements..

| **Group**  **no** | **Food Group** | **USDA Recommended Intake** | **Notes** | **WWEIA codes** |
| --- | --- | --- | --- | --- |
| 1 | Dark-green vegetables | 1 1/2 cup eq/wk | fresh, frozen, canned | wweia cat = 6408 ‘Dark green vegetables’ & Na^+^ < 250 mg |
| 2 | Red/orange vegetables | 5 1/2 cup eq/wk | fresh, frozen, canned, juice | wweia cat in (6402 ‘Tomatoes’, 6404 ‘Carrots’, 6406 ‘Other red and orange vegetables’) & Na^+^ < 250 mg |
| 3 | Legumes (beans&peas) | 1 1/2 cup eq/wk | dry or canned | wweia cat = 2802 ‘Beans, peas, legumes’ & Na^+^ < 250 mg |
| 4 | Starchy vegetables | 5 cup eq/wk | fresh, frozen, canned | wweia cat in (6418 ‘Other starchy vegetables’, 6802 ‘White potatoes baked or broiled’, 6806 ‘Mashed potatoes and white potato mixtures’) & Na^+^ < 250 mg |
| 5 | Other vegetables | 5 cup eq/wk | fresh, frozen, canned | other vegetables in wweia main group vegetables & Na^+^ < 250 mg |
| 6 | Fruits | 2 cup eq/d | fresh, frozen, canned, dried, juice | wweia main group fruit or subgroup = 70 ‘100% Juice’ |
| 7 | Whole grains | 3 oz eq/d | products & ingredients | wweia main group = grains, g_whole > 0 & g_whole/g_total >= .6 & d_total = 0 & Na^+^ < 300 mg |
| 8 | Refined grains | 3 oz eq/d | products & ingredients | wweia main group = grains, g_refined > 0 and g_whole/g_total < .6 & d_total = 0 & Na^+^ < 300 mg |
| 9 | Dairy | 3 cup eq/d | milk, yogurt, cheese, soymilk; lowfat; no creams | (wweia main group = dairy & total fat < 1.5g) or (subgroup = 16 ‘Cheese’ & total fat < 20g & Na^+^ < 600mg) |
| 10 | Seafood | 8 oz eq/wk |  | wweia subgroup = seafood |
| 11 | Meats, poultry, eggs | 26 oz eq/wk | lean | ((wweia cat in (2002 ‘Beef excludes ground’, 2004 ‘Ground beef’, 2006 ‘Pork’, 2202 ‘Chicken, whole pieces’, 2204 ‘Chicken patties, nuggets and tenders’, 2206 ‘Turkey, duck, other poultry’) & Na^+^ < 500mg) or (wweia cat = 2502 ‘Eggs and omelets’ & Na^+^ < 300mg)) & total fat < 15mg & cholesterol < 300mg & v_redor_total = 0 |
| 12 | Nuts, seeds, soy | 5 oz eq/wk | unsalted | ((wweia cat = 2804 ‘Nuts and seeds’ & Na^+^ = 0) or (wweia cat = 2806 ‘Processed soy products’ & Na^+^ < 300mg)) & saturated fat < 10 g & V-redor_total = 0 |
| 13 | Oils | 27 g/d |  | wweia cat in (8002 ‘Butter and animal fats’, 8004 ‘Margarine’, 8010 ‘Mayonnaise’, 8012 “Salad dressing and vegetable oils’) & fped oils > 90 g & fped solid_fats = 0g |
| 14 | Other foods | 270 kcal/d |  | wweia main group in (3 ‘Mixed dishes’, 5 ‘Snacks and sweets’ ,8 ‘Beverages nonalcoholic’, 9 ‘Alcoholic beverages’, 13 ‘Sugars’) & Na^+^ < 250mg & fped added sugars = 0g |
