# Supplementary material for: An Economic Gap Between the Recommended Healthy Food Patterns and Existing Diets of Minority Groups in the US National Health and Nutrition Examination Survey 2013–14
Source: Front Nutr. 2019 Apr 4;6:37. doi: 10.3389/fnut.2019.00037 (PMC6458255; doi:10.3389/fnut.2019.00037)
Supplement: Supplementary file 2 [file Data_Sheet_2.docx]

**Appendix Table A2**. Estimated amounts of food groups in the National Health and Nutrition Examination Survey (NHANES 2013-14) and in the recommended USDA Healthy Eating Patterns: Vealthy Vegetarian, Healthy US Style, and Healthy Mediterranean.

|  | Observed | | Vegetarian | | Healthy US | | Mediterranean | |
| --- | --- | --- | --- | --- | --- | --- | --- | --- |
|  |  |  |  |  |  |  |  |  |
| Vegetables, total (cup-eq) | 1.32 |  | 2.77 |  | 2.91 |  | 2.71 |  |
| Vegetables, starchy (cup eq) | 0.38 |  | 0.91 |  | 0.91 |  | 0.87 |  |
| Vegetables, dark green (cup eq) | 0.13 |  | 0.34 |  | 0.37 |  | 0.34 |  |
| Vegetables, red orange (cup eq) | 0.35 |  | 0.83 |  | 0.85 |  | 0.85 |  |
| Other vegetables (cup eq) | 0.46 |  | 0.69 |  | 0.78 |  | 0.64 |  |
| Legumes (beans & peas) | 0.10 |  | 0.43 |  | 0.24 |  | 0.24 |  |
| Fruits, total (cup eq) | 0.92 |  | 2.11 |  | 2.41 |  | 2.92 |  |
| Fruits, whole (cup eq) | 0.63 |  | 2.04 |  | 2.01 |  | 2.50 |  |
| Grains, whole (oz eq) | 0.81 |  | 3.71 |  | 3.06 |  | 3.06 |  |
| Grains, refined (oz eq) | 5.50 |  | 3.70 |  | 2.46 |  | 2.46 |  |
| Dairy, total (cup eq) | 1.67 |  | 3.24 |  | 3.09 |  | 2.09 |  |
| Yogurt (cup eq) | 0.06 |  | 0.20 |  | 0.20 |  | 0.13 |  |
| Cheese (cup eq) | 0.77 |  | 0.26 |  | 0.15 |  | 0.11 |  |
| Protein foods, total (oz eq) | 5.65 |  | 2.70 |  | 5.77 |  | 6.78 |  |
| Seafood (oz eq) | 0.43 |  | 0 |  | 0.71 |  | 1.29 |  |
| Nuts, seeds (oz eq) | 0.69 |  | 1.08 |  | 0.43 |  | 0.43 |  |
| Soy products (oz eq) | 0.07 |  | 1.15 |  | 0.30 |  | 0.31 |  |
| Oils (g) | 24.1 |  | 26.8 |  | 27.6 |  | 30.9 |  |
| Solid fats (g) | 35.0 |  | 9.1 |  | 7.42 |  | 6.65 |  |
| Added sugars (tsp) | 16.7 |  | 3.56 |  | 0 |  | 0 |  |
|  |  |  |  |  |  |  |  |  |
|  |  |  |  |  |  |  |  |  |
